# Unveiling and harnessing the human gut microbiome in the rising burden of non-communicable diseases during urbanization

**DOI:** 10.1080/19490976.2023.2237645

**Published:** 2023-07-27

**Authors:** Ziyu Huang, Yue Li, Heekuk Park, Martin Ho, Kanchan Bhardwaj, Naoki Sugimura, Hye Won Lee, Huicui Meng, Matthias P. Ebert, Kang Chao, Elke Burgermeister, Aadra P. Bhatt, Sudarshan A. Shetty, Kai Li, Weiping Wen, Tao Zuo

**Affiliations:** a Key Laboratory of Human Microbiome and Chronic Diseases, Sun Yat-Sen University, Ministry of Education, Guangzhou, China; b Guangdong Institute of Gastroenterology, the Sixth Affiliated Hospital, Sun Yat-Sen University, Guangzhou, China; c Biomedical Innovation Centre, the Sixth Affiliated Hospital, Sun Yat-Sen University, Guangzhou, China; d The Sixth Affiliated Hospital, Sun Yat-Sen University, Guangzhou, China; e Department of Medicine, Division of Infectious Diseases, Columbia University Irving Medical Centre, New York, NY, USA; f Department of Engineering, University of Cambridge, Cambridge, UK; g Department of Biotechnology, Faculty of Engineering and Technology, Manav Rachna International Institute of Research and Studies, Haryana, India; h Gastrointestinal Centre and Institute of Minimally-Invasive Endoscopic Care (iMEC), Sano Hospital, Kobe, Japan; i Institute of Gastroenterology and Department of Internal Medicine, Yonsei University College of Medicine, Seoul, Korea; j School of Public Health (Shenzhen), Shenzhen Campus of Sun Yat-Sen University, Sun Yat-Sen University, Shenzhen, China; k Guangdong Provincial Key Laboratory of Food, Nutrition and Health, Guangzhou, China; l Guangdong Province Engineering Laboratory for Nutrition Translation, Guangzhou, China; m Department of Medicine II, Medical Faculty Mannheim, Heidelberg University, Mannheim, Germany; n DKFZ-Hector Cancer Institute, Mannheim, Germany; o Mannheim Cancer Centre (MCC), University Medical Centre Mannheim, Mannheim, Germany; p Department of Gastroenterology, the Sixth Affiliated Hospital, Sun Yat-Sen University, Guangzhou, China; q Department of Medicine, Centre for Gastrointestinal Biology and Disease, and the Lineberger Comprehensive Cancer Centre, University of North Carolina, Chapel Hill, NC, USA; r Department of Medical Microbiology and Infection Prevention, University Medical Centre Groningen, Groningen, The Netherlands

**Keywords:** the gut microbiome, non-communicable diseases, urbanization, fecal microbiota transplantation, diet

## Abstract

The world is witnessing a global increase in the urban population, particularly in developing Asian and African countries. Concomitantly, the global burden of non-communicable diseases (NCDs) is rising, markedly associated with the changing landscape of lifestyle and environment during urbanization. Accumulating studies have revealed the role of the gut microbiome in regulating the immune and metabolic homeostasis of the host, which potentially bridges external factors to the host (patho-)physiology. In this review, we discuss the rising incidences of NCDs during urbanization and their links to the compositional and functional dysbiosis of the gut microbiome. In particular, we elucidate the effects of urbanization-associated factors (hygiene/pollution, urbanized diet, lifestyles, the use of antibiotics, and early life exposure) on the gut microbiome underlying the pathogenesis of NCDs. We also discuss the potential and feasibility of microbiome-inspired and microbiome-targeted approaches as novel avenues to counteract NCDs, including fecal microbiota transplantation, diet modulation, probiotics, postbiotics, synbiotics, celobiotics, and precision antibiotics.

## Introduction

Non-communicable diseases (NCDs) are a class of chronic diseases that do not transmit directly from one person to another, including metabolic diseases, autoimmune diseases, cancers, and mental illnesses. For a long time, NCDs were considered a burden of the developed world.^1^ However, with the rapid increase of global urbanization over the past several decades, manifesting in a mass migration of populations moved from rural to urban areas,^1,2^ accumulating evidence shows that substantial disease burdens of NCDs are emerging not only in high-income countries but also in low- and middle-income countries.^1,3^ Indeed, NCDs have been rising and are among the leading causes of death worldwide, especially in developing countries and newly urbanized countries.^4^ It is estimated that over 68% of the world population will be living in urban areas by 2050,^2^ presenting an urgent need to understand how NCDs manifest through urbanization and how to counteract such diseases.

**Figure 1.**
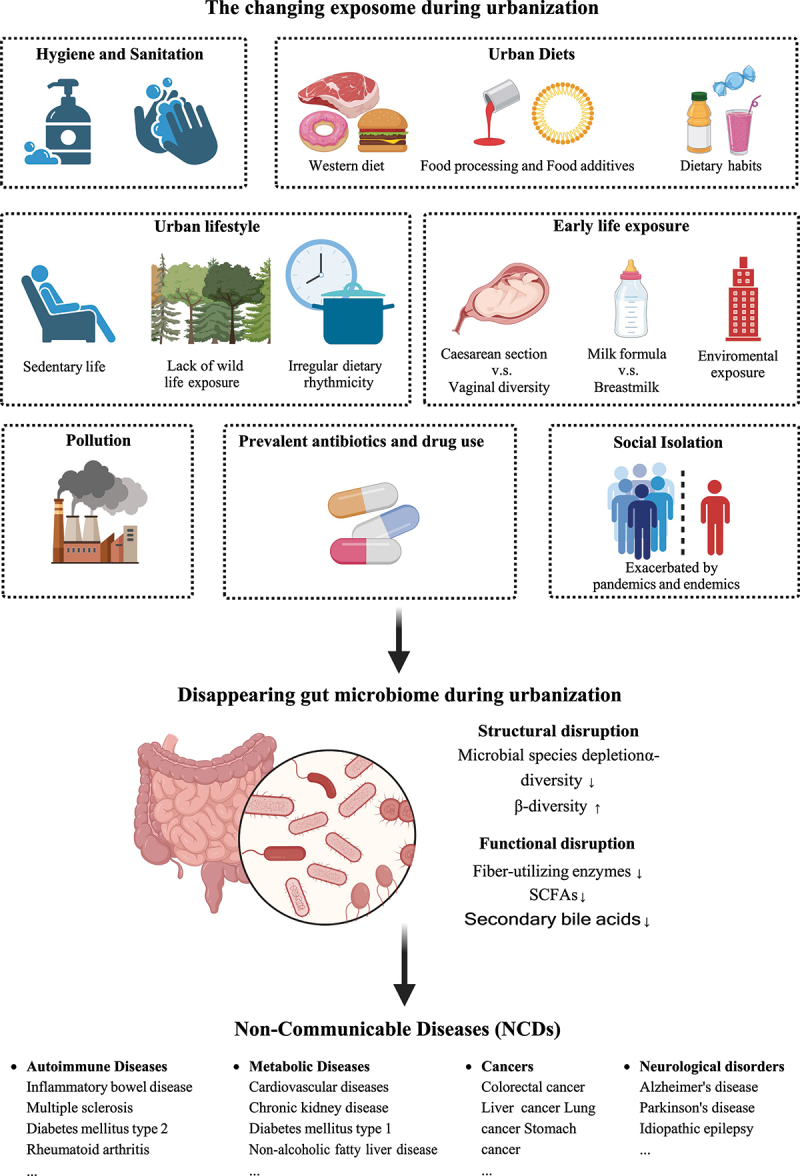
The changing exposome during urbanization is linked to an impaired gut microbiome assembly and non-communicable diseases.

Epidemiological studies have shown that shifts in environment, diet, and lifestyle during urbanization are closely related to the pathogenesis of NCDs.^4^ Meanwhile, a growing body of basic and translational evidence corroborates that these host-external factors can influence the configuration and function of the gut microbiome, alluding to causal relationships among urbanization, the gut microbiome, and the onset and progression of NCDs^5,6^, though presently a substantial amount of evidence is correlative, while relatively less evidence is causative in nature. The large collection of microbes (bacteria, fungi, archaea, and viruses/phages), and their genes in the gastrointestinal (GI) tract of the human host are referred to as the gut microbiome. A healthy gut microbiome is featured by a compositionally and functionally diverse collection of stable, core microbes without overt pathogenic microbes.^7^ Commensal gut microbes and their products make substantial contributions to host health, by calibrating the systemic immune and metabolic functions of the host as well as the microenvironment homeostasis within diverse niches of the human body.^8^ A large number of studies have shown that urbanization is associated with decreased microbial species diversity within a person’s gut microbiome (α diversity) and an increased dissimilarity between the gut microbiome of persons (β diversity); this phenomenon is referred to as the “disappearing gut microbiome during urbanization” (Figure 1).^9,10^ These alterations to the human gut microbiome are hypothesized to be a culprit in the rising NCD incidences. The gut microbiome of humans is immensely influenced by urbanization-associated, external factors, such as pollution, diet, and lifestyles (Figure 1). Alterations in these factors during urbanization can evoke a dysbiotic shift in the gut microbiome, changing intestinal permeability and resulting in dysfunctional metabolism and immunity, the effect of which may extend to extra-intestinal organs.^11,12^ The chronic inflammation or mal-functional state of the host caused by an imbalance of the gut microbiome may lead to the onset of NCDs.^13,14^ An understanding of the tripartite interaction among the urbanized environment, human gut microbiome, and NCD pathogenesis would illuminate preventive and therapeutic avenues for contemporary diseases.

## The increasing global burden of NCDs with urbanization

Urbanization refers to the shift in population from rural to urban settlements and the corresponding increase in the population size and density in urban areas.^15^ In 1990, approximately 43% of the global population lived in urban areas; by 2021, the share of the urban population increased to nearly 57%.^2^ The incidence of NCDs mirrors the urbanization trend, establishing themselves as the leading cause of death globally.

**Figure 2.**
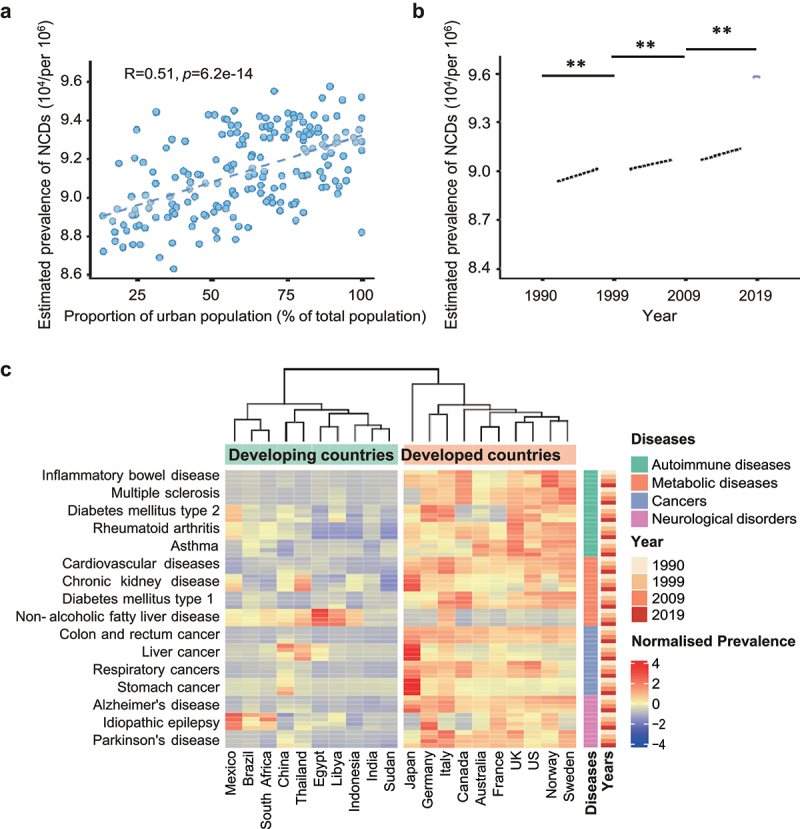
The rising global and national burdens of NCDs with urbanization.

To gain a global view of the disease burden of NCDs and assess its relationship with urbanization, we obtained the estimated prevalence data of NCDs (per 100,000 individuals) from the Global Burden of Disease Study (GBD) 2019^16^ and evaluated its correlation with urbanization levels (defined by the proportion of the urban population, a surrogate of urbanization) retrieved from the UN World Urbanization Prospects database^17^, stratified by countries (developing versus developed countries) and by the last 30 years at 10-year intervals (Figure 2). A total of 189 countries and 174 NCDs were included in the analysis. Overall, the estimated prevalence of NCDs was significantly associated with the proportion of the urban population across different countries (cross-sectional data in the year 2019, pooled Spearman’s rank correlation coefficient *r* = 0.51, *p* < 0.0001, Figure 2A). Through a time-course analysis along the chronological axis, we observed a time-dependent global increase in the estimated prevalence of NCDs over the past 30 years (from 1990 to 2019, Figure 2B). These data indicated that the prevalence of NCDs was correlated with both the urbanization level cross-sectionally and the development trajectory of the world longitudinally.

A. The correlation between the estimated prevalence of NCDs (per 100,000) and the proportion of urban population (% of total population) across 189 countries in 2019. Correlation coefficient and statistical significance were calculated using Spearman’s rank correlation by *R* cor function. NCDs: non-communicable diseases.

B. The estimated prevalence of NCDs in 189 countries and territories along the chronological years (1990, 1999, 2009, 2019). *P < 0.05; **P < 0.01; ***P < 0.001 determined by one-way ANOVA.

C. 16 prevalent NCDs and 20 countries were selected for visualization of disease prevalence in relation to urbanization (developing versus developed countries, along the chronological years from 1990 to 2019). The estimated prevalence values for each NCD at the indicated year were normalized based on the mean and standard deviation by *R* scale function. The estimated prevalence levels of NCDs are shown as a heatmap.

For a more granular view of the impact of urbanization on NCDs across different countries, we stratified the estimated prevalence levels of 16 prevalent NCDs into four classes (metabolic diseases, autoimmune diseases, cancers, and mental illnesses) across 10 developing countries: China, India, Mexico, Brazil, South Africa, Thailand, Libya, Egypt, Indonesia, Sudan; and 10 developed countries: US, UK, Japan, Germany, France, Australia, Norway, Sweden, Canada, Italy (from 1990 to 2019, Figure 2C). The result showed that the prevalence of nearly all surveyed NCDs was remarkably higher in developed countries than in developing countries, except for nonalcoholic fatty liver diseases (NAFLD) (Figure 2C). From 1990 to 2019, the prevalence of NCDs increased across developing countries, particularly inflammatory bowel disease (IBD), chronic kidney disease, type 2 diabetes mellitus (T2DM), Alzheimer’s disease, and Parkinson’s disease (Figure 2C). Overall, NCDs are substantially prevalent in developed countries and increasing in most developing countries. Among the NCDs, cardiovascular diseases (CVD), cancers, chronic respiratory diseases, and diabetes are the top four killers, the mortalities of which are still on the rise annually.^18^ China is one of the fastest-growing major economies in the world exhibiting rapid urbanization, the rate of urban population increased from 26.4% in 1990 to 60.3% in 2019, where the estimated prevalence of IBD rose from 20.2 (95%CI 16.8–24.1) per 100,000 individuals to 64.1 (95%CI 54.6–75.2) per 100,000 individuals. Beyond developing countries, the share of NCDs increased from 1990 to 2019 in almost all countries except for Ukraine and Lesotho.^16^ The changing landscape of NCDs prevalence in recent 30 years cannot be ascribed to genetics as the genetic makeup of humans cannot change much across one generation. Alternatively, lifestyle and environmental changes accompanying the transition from a rural to an urban settlement are most likely contributing to the rising epidemiology of NCDs, especially considering the enhanced risk for NCDs in children of immigrants during urbanization.

## The contributing role of gut microbiome under the influence of urbanization to NCDs

As a cornerstone of human health, the gut microbiome is strongly influenced by host-external factors and downstream extensively regulating host physiology. Demonstrating the former, immigration from a developing country to a developed country results in a loss of the gut microbiome diversity and function, such as the depletion of propionate producers *Prevotella* spp. and their glycoside hydrolases for fiber degradation.^19^ This microbial depletion increased with a longer duration of urban residence and was even compounded across generations in humans.^19^ The structural and/or functional disruption of the gut microbiome by urbanization factors, generally featured by a richness-depleted microbial ecology (particularly depletion of rare and salutary microbial species) and blooms of opportunistic pathogens in the gut, contribute largely to the pathogenesis and course of NCDs (Figure 1, Table 1). The increased hygiene practice and cleanliness, adopted in urbanized societies, significantly limit horizontal microbial transmission (herein person-to-person microbial transmission between members that are not in a parent–progeny relationship, a critical event for diversifying and maintaining the gut microbial taxa and functions).^39,40^ Such lifestyle change is also proposed to be a critical contributor to the rise of NCDs, as the gut microbiomes in most patients with NCDs are characterized by a greater between-individual difference than between healthy individuals;^9,41^ this parallels the Anna Karenina principle in the gut microbiome field that “all happy families look-alike; each unhappy family is unhappy in its own way”.^42^ In favor of this principle, our recent studies also indicate that higher β diversity of the gut microbiome was found in urban residents than rural residents and that rural residents have more rare, probiotic microbial species in abundance in their gut.^43^ Altogether, the changing landscape of exposome (refers to the measure of all exposures from both external and internal sources, including chemical, physical, biological, and social factors, from conception onward, over a complete lifetime)^44^ during urbanization may carry out detrimental roles in the gut microbiome linking to NCDs. Therefore, it is important to elucidate how urban exposome impacts the gut microbiome underpinning the changing epidemiology of NCDs.

**Table 1.** Studies on the effects of urbanization-associated factors on the gut microbiome in relation to host health.

UrbanisaUrbanization-associated factors	Host	Alteration in the gut microbiome		Effects on host immunity/metabolism	NCDs potentially caused by the altered gut microbiome	Ref.
		Increase taxon	Decrease taxon			
**Environmental factors**						
PM 2.5	Mouse	f_Lachnospiraceae f_Rikenellaceae g_*Marvinbryanatia* s_*Alistipes finegoldii*	Bacterial richness c_Epsilonproteobacteria o_Campylobacterales f_Helicobacteraceae f_Peptostreptococcaceae f_Clostriiaceae g_*Romboutsia* g_*Papillibacter* g_*Allobaculum* g_*Turicibacter*	Glucose intolerance Insulin resistance	Diabetes	^20^
PM 10	Mouse	p_Firmicutes p_Verrucomicrobia	p_Bacteroidota	TNF-α, CXCL-1, IL-1β, IL-12, IL-13, IL-17, IL-10 ↑ IFN-γ and IL-2 *↓* Isovalerate and isobutyrate ↑ Butyrate and valerate *↓*	Acute and chronic intestinal inflammatory	^21^
PM2.5 and PM1	Human	-	α diversity p_Firmicutes p_Proteobacteria p_Verrucomicrobia	Impaired fasting glucose	T2DM	^5^
Nitrogen oxides	Human	f_Coriobacteriaceae	f_Bacteroidaceae	Impaired fasting glucose	T2DM and obesity	^22^
**Hygiene factors**						
Lipids and lipid-like molecules from cleaning products and detergents	Human	g_*Candida* g_*Aspergillus*	α diversity g_*Debaryomyces* g_*Saccharomyces* g_*Trichosporon* g_*Fusarium*	-	-	^23^
**Dietary factors**						
Western diet	Human	β diversity g_*Alistipes* g_*Bilophila* g_*Bacteroides*	g_*Roseburia* s_*Eubacterium rectale* s_*Ruminococcus bromii*	Colonic deoxycholic concentrations ↑ Colonic H_2_S ↑	CRC and IBD	^24^
		g_*Prevotella*	g_*Bacteroides*	Fecal tryptophan *↓*	Obesity	^25^
		-	s_*Eubacterium rectale* s_*Clostridium symbiosum* s_*Oscillospira guillermondii* s_*Roseburia intestinalis*	Secondary bile acids ↑ Colonic butyrate *↓* TMAO ↑	CRC and CVD	^26^
	Mouse	c_Erysipelotrichi c_Bacilli s_*Clostridium innocuum* s_*Eubacterium dolichum*, s_*Catenibacterium mitsuokai*	p_Bacteroidota	-	Obesity	^27^
Choline and carnitine in red meats and processed meats	Human	f_Peptostreptococcaceae f_Clostridiaceae g_*Prevotella* g_*Clostridium*	g_*Lachnospira*	TMA ↑ TMAO ↑	CVD	^28^
Heme in red meats	Mouse	p_Bacteroidota p_Proteobacteria p_Verrucomicrobia s_*Akkermansia muciniphila*	-	Colonic mucus barrier integrity *↓* Fecal trisulfide ↑	CRC	^29^
Dietary emulsifiers (carboxymethylcellulose and polysorbate-80)	Mouse	p_Proteobacteria p_Verrucomicrobia s_*Ruminococcus gnavus* s_*Akkermansia muciniphila*	o_Bacteroidales	Colonic mucus barrier integrity *↓* Fasting glucose ↑ Fecal level of butyrate *↓*	Obesity Metabolic syndrome Colitis	^30^
Artificial sweeteners	Human	s_*Prevotella copri* s_*Bacteroides xylanisolvens* s_*Bacteroides coprophilus* s_*Parabacteroides goldsteinii* s_*Lachnospira* spp.	-	Serum level of serine, N-acetyl alanine, aspartate, quinolinate, cystine, lysine, and glycyl-L-valine ↑	Glucose intolerance	^31^
	Mouse	g_*Anaeroplasma* g_*Parabacteroides* s_*Bacteroides acidifaciens*	o_Clostridiales g_*Clostridium* s_*Lactobacillus reuteri*	Fecal level of propionate and acetate ↑	Glucose intolerance	^32^
**Urban lifestyles**						
Sedentary lifestyle	Human	f_Enterobacteriaceae o_Enterobacteriales	f_Paraprevotellaceae f_Lachnospiraceae g_*Lachnospira*	SCFA *↓*	Obesity	^33^
**Prevalent antibiotic use**						
Vancomycin	Human	p_Proteobacteria g_*Haemophilus* g_*Serratia* s_*Escherichia coli*	α diversity p_Firmicutes *Clostridium cluster IV/XIVa* s_*Lactobacillus plantarum* s_*Faecalibacterium prausnitzii* s_*Eubacterium hallii*	Primary bile acids ↑ Secondary bile acids *↓*	Insulin sensitivity *↓* T2DM	^6^
Ampicillin, bacitracin, meropenem, neomycin, vancomycin	Mouse	-	Eradication of most of commensal bacteria	Acetate, Butyrate, Propionate *↓* TMA *↓* Adenine *↓* Uracil *↓* Brain-derived neurotrophic factor *↓* N-methyl-d-aspartate receptor subunit 2B ↑ Serotonin transporter ↑ Neuropeptide ↑	Cognitive deficit	^34^
Tylosin, amoxicillin	Mouse	f_Prevotellaceae f_Enterobacteriaceae s_*Clostridium citroniae*	Microbial richness and evenness	Treg *↓* TNF-α ↑ IL-22 *↓* Muc2 *↓*	IBD	^35^
**Early life exposure**						
Cesarean section	Human	-	g_*Bifidobacterium* g_*Bacteroides* g_*Lactobacillus* g_*Enterobacter* g_*Haemophilus* g_*Staphylococcus* g_*Streptococcus*	Treg *↓*	Asthma	^36,37^
Milk formula	Human	s_*Clostridium difficile* s_*Granulicatella adiacens* s_*Citrobacter* spp. s_*Enterobacter cloacae* s_*Bilophila wadsworthia*	s_*Lactobacillus johnsonii* s_*Lactobacillus gasseri* s_*Lactobacillus paracasei* s_*Lactobacillus casei* s_*Bifidobacterium longum*	-	-	^38^

Abbreviation: PM, particulate matters; TNF, tumor necrosis factor; CXCL, the chemokine (C-X-C motif) ligand; IL, interleukin; IFN, interferon; H_2_S, hydrogen sulfide; CRC, colorectal cancer; IBD, inflammatory bowel disease; TMAO, trimethylamine N-oxide; CVD, cardiovascular diseases; TMA, trimethylamine; SCFA, short-chain fatty acid; Treg, regulatory T cell.

## Environmental pollution

Urbanization is inevitably casting tremendous pressure on the environment due to increased industrialization and population size.^45^ Air pollution, which is collectively a mixture of gases containing volatile organic compounds and particulate matters (PM), represents one of the consequent events during urbanization.^46^ Epidemiologic studies indicated that PM2.5 (fine particles with a diameter of 2.5 μm or less) was a significant risk factor for the T2DM burden globally.^5,22^ In addition, recent studies underpinned a mediating effect of the gut microbiome between PM2.5 and T2DM,^5^ since PM may reach the intestine through respiration-circulation and ingestion followed by casting an effect on the gut microbiome.^47^ A community-based cross-sectional study conducted in the Chinese population (*n* = 6627) found that PM2.5 was positively associated with the risks of impaired fasting glucose or T2DM and negatively associated with the α diversity of the gut microbiota.^5^ Another epidemiological study conducted in Southern California in the US (*n* = 43) also suggested that the positive correlation between air pollutants and fasting glucose levels can be explained by the decrease of Bacteroidaceae and the increase of Coriobacteriaceae.^22^ Of them, Coriobacteriaceae is a main producer of phenylacetylglutamine in the gut,^48^ which was recently identified as a biomarker of T2DM.^49^ Prolonged exposure to PM could also cause changes in levels of short-chain fatty acids (SCFAs, derived from gut microbial fermentation) in the cecum of mice, resulting in increased isobutyrate and decreased butyrate.^21^ This alteration in SCFAs production was found to suppress insulin secretion and islet β-cells functioning.^11^ These findings highlight that ingestion of air pollutants can substantially alter the gut microbiome and affect the metabolic functions of the host. Akin to the effect of environmental pollution on the gut microbiome, environmental pollution also has a great impact and direct link to respiratory infections and asthma via its direct effect on the respiratory microbiome.^50,51^

Urban and rural areas are distinct in indoor chemical and microbial environments, leading to different exposure profiles for their human residents.^23^ This environmental discrepancy nurtures different gut microbiomes. A recent study, spanning several areas of the same latitude in the Amazon rainforest, detected large quantities of lipids and lipid-like molecules from cleaning products and detergents on urban house surfaces.^23^ Chemicals derived from medications, including the beta blocker metoprolol and the antifungals ketoconazole and clotrimazole, were only detected in more urbanized regions.^23^ House surface chemicals in urban areas were associated with a lower diversity of environmental microbiomes.^23,52^ As a critical source of human gut microbes, the decrease in environmental microbiome diversity may directly reduce exposure and subsequent colonization of microbes in the human gut. In favor of this hypothesis, the fecal and anal microbiome of humans was found to be concomitantly decreased with the altered environmental microbiome in urban areas.^23,52^ Overall, changes in the environment during urbanization lead to reduced exposure to natural microbes and enhanced exposure to sanitation chemicals, which may consequently lead to a decreased α diversity of the gut microbiota yet increased β diversity – a predisposition to NCDs.

## Urbanized dietary habits

**Figure 3.**
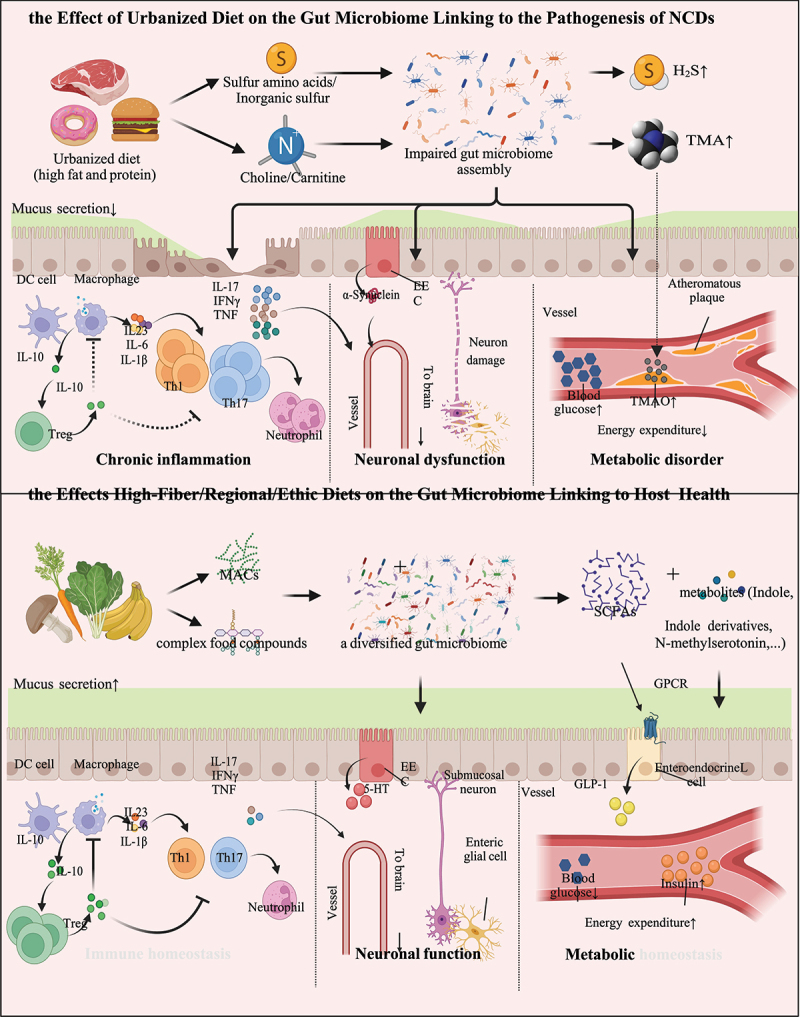
Impact of diets on the gut microbiome underlying the pathogenesis of NCDs.

Diet is one of the most influential factors that directly shape the human gut microbiome (Figure 3). Despite baseline dietary habits varying substantially across geography and ethnicity, there is a converging and universal transition from traditional characteristic diets (regional/ethnic) to a western diet style worldwide during urbanization.^64^ Western diet is characterized by high intakes of saturated fats, processed meats, added sugars, and refined grains; and low intakes of fibers, fruits, vegetables, and fish.^65^ Western diet is extensively investigated in humans and mice and has been found to deplete gut microbial species (including loss of multiple beneficial commensal taxa *Prevotella* spp., *Roseburia* spp., and *Ruminococcus bromii*), resulting in a decrease in the α diversity of the gut microbiome.^24^ These changes to the gut microbiome elicited by western diet have been causally linked to NCDs in mice, such as metabolic diseases and autoimmune diseases,^66^ and are epidemiologically associated with enhanced risks and disease outcomes of NCDs in humans.^25,67^

During urbanization, a variety of urbanization-associated factors induce the structural and compositional dysbiosis of the gut microbiome. The sulfur compounds and Choline/Carnitine in urban diets are metabolized into hydrogen sulfide and TMA, respectively, by the gut microbes.^28,53^ The absorbed TMA is subsequently oxidized to TMAO in the liver.^54^ Such reactions mediated by the gut microbiome pose risks to CRCs and CVDs.^41,54^The influx of a large number of microbes and their metabolites through the dampened gut barrier due to urban lifestyles and western diet induces the proliferation of pro-inflammatory immune cells (such as Th1 and Th17 cells) and the release of pro-inflammatory cytokines (such as IL-6, IL-17, IFN-γ, and TNF).^55,56^ The inflammatory state and pathogens mediate demyelination, axonal damage, and neurodegeneration, leading to multiple sclerosis.^57^ The inflammation also enhances α-synuclein expression and misfolding, leading to Parkinson’s disease.^58^ By contrast, high-fiber and certain regional and ethnic diets cultivate a diversified gut microbiome, which can metabolize MACs and food ingredients to an array of beneficial compounds and celobiotics, such as SCFAs and *N*-methylserotonin.^59,60^ Such diversified gut microbiota and their metabolites strengthen the gut barrier function of the host by stimulating a thick mucus layer and activating a proper immune profile to counteract the invasion of pathogens.^61^ Under this circumstance, Treg cells suppress pro-inflammatory cells and prevent autoimmune diseases.^62^ A proper immune profile together with a steady secretion of gut hormones by enteroendocrine cells and a balanced enteric nervous system mediates mutualistic communication between the gut microbiome and the brain.^58^ SCFAs regulate enteroendocrine L cells to release GLP-1, which reduces the level of blood glucose and increase the secretion of insulin.^63^DC, dendritic cell; EEC, enteroendocrine cell; FFAR, free fatty acid receptor; GLP-1, Glucagon-like peptide-1; IFN-γ: interferon-γ; IL, interleukin; MACs, microbiota-accessible carbohydrates; NCDs, non-communicable diseases; SCFAs, short-chain fatty acids; Th, T helper cell; TMA, trimethylamine; TMAO, trimethylamine N-oxide; Treg, regulatory T cell; TNF, tumor necrosis factor; 5-HT, 5-hydroxytryptamine.

Among various dietary components, dietary fibers have the most intimate relationship with the gut microbiome, since they contain a variety of microbiota-accessible carbohydrates (MACs) that are indigestible by the human host, serving as the direct supply of nutrients for the gut microbiome. Western diet and modern food processing technologies have resulted in a significant deficiency in MACs in foods. A recent murine study showed that the low-fiber diet-induced gut microbial extinction (particularly low-abundant taxa from the Bacteroidales order) was irreversible and microbial extinction compounded in the offspring of the affected mice.^68^ In addition, the low-MACs diet can significantly impact the metabolic pathways of gut microbiome, especially mucus-degrading bacteria, such as *Bacteroides thetaiotaomicron* and *Akkermansia muciniphila*. *B. thetaiotaomicron*, as nutritional generalists in the gut, can metabolize dietary polysaccharides and mucus glycan as carbon sources. However, they may turn to degrade and metabolize host mucus glycans, under conditions of shortage in dietary polysaccharides, resulting in compromising colonization resistance to pathogens and increasing susceptibility to infection and colitis.^69^

A same bacterium (such as *A. muciniphila*) may play opposing roles in regulating disease susceptibility, depending on the niche context (external factor) and microorganismal activity (internal factor) at play. Under dietary fiber-depleted circumstances, the gut niche is open for *A. muciniphila* to blossom and to utilize the mucosal mucin glycans, resulting in encroached intestinal mucosa and exacerbated intestinal inflammation; in this case, the detrimental role of *A. muciniphila* is largely ascribed to its mucin-degrading activity in the context of fiber-depletion.^69^ On the other hand, proteins produced by *A. muciniphila* (such as Amuc_1100 and Amuc_2172) are protective against obesity, diabetes, colitis, and its associated tumorigenesis;^70–72^ in this case, the protective role of *A. muciniphila* is largely ascribed to its biosynthesis activity of salutary molecules. Hence, the virtual role of such controversial microorganisms in disease susceptibility is hinged on nutrient availability, colonization resistance, and importantly a balance between its various yet opposing bioactivities. Moreover, it is important to note that while dietary fiber supplements may reduce degradation of the intestinal mucus layer by rescuing gut microbes, purified prebiotic fibers produced by modern food processing technologies do not achieve such an effect.^69^ Therefore, maintaining the originality and complexity of MACs in diets is important for boosting a healthy gut microbiome as well as host health. In addition, MACs are the substrates of microbial fermentation, the absence of which has a direct deleterious effect on the production of short-chain fatty acids (SCFAs), potentially leading to counteract IBD, metabolic diseases, allergic diseases, cancers, and neuronal dysfunction.^73–76^ These studies suggest that fiber deficiency and western diet, as an urbanized dietary habit, evoke an irreversible impairment to the gut microbiome diversity and functionality.

Chronic ingestion of red meats and processed meats has been reported to increase plasma and urine levels of trimethylamine N-oxide (TMAO), a molecule associated with gut microbial metabolism and an increased risk of CVD^41,54^ in a randomized controlled trial (RCT, *n* = 113).^12^ Gut microorganisms metabolize high amounts of choline and carnitine in red meats and processed meats to trimethylamine (TMA).^28^ Once absorbed via the GI tract, TMA is delivered to the liver and oxidized to TMAO, which then promotes atherosclerosis, thrombosis, heart failure via augmented macrophage cholesterol accumulation and foam cell formation.^54^ Heme, another component of red meat, can increase the abundance of sulfate-reducing bacteria, which can lyse mucin polymers.^29^ With the disruption of mucus barrier integrity, the cytotoxic heme reaches to and injures the colonic epithelial surface cells, resulting in epithelial hyperproliferation and inhibition of apoptosis, which can eventually develop into CRC.^29^ High saturated fats in processed foods and meat products are also associated with CRC pathogenesis.^77^ To emulsify a large amount of dietary fat, the liver increases the secretion of bile acids. When reaching the colon, bile acids are metabolized into secondary bile acids by the anaerobic bacteria (*Clostridium* cluster XIVa and XI, and *Eubacterium*).^78^ Of secondary bile acids, deoxycholic acid is a mutagen and has been shown to elicit DNA damage and apoptosis resistance by causing oxidative stress, elevating the risk of proximal colon cancer.^78^

The food industry in urbanized societies is rapidly expanding the use of food additives, such as emulsifiers, preservatives, and artificial sweeteners, which have all been shown to perturb the host homeostasis (both in the gut and systemically) by disrupting the membership and functions of the gut microbiota.^79^ Carboxymethylcellulose and polysorbate-80, two common emulsifiers, were shown to aggravate colitis in interleukin (IL)-10^−/−^ mice and induce intestinal inflammation and obesity/metabolic syndrome in wild-type mice by damaging the intestinal multi-layered mucus structure and gut microbiome composition.^30^ A latest RCT study in healthy volunteers (*n* = 120) showed that the artificial sweeteners saccharin and sucralose altered the gut microbiome functionally and led to varying degrees of impairment of glucose tolerance compared to the placebo group.^31^ The fecal microbiomes from top or bottom human responders of sweeteners were then transplanted into germ-free (GF) mice, where the glycemic responses in recipient mice largely reflected those noted in their corresponding human donors.^31^ This suggests that the dampened gut microbiome plays a causal role in poor glycemic responses. In murine models, artificial sweeteners enhance glycan degradation to various products in the gut microbiome, of which propionate and acetate are precursors and signaling molecules for *de novo* glucose and lipid synthesis by the host, inducing a propensity to obesity and glucose intolerance.^32^ These studies together established a causal relationship between food additives commonly consumed in urban communities and risks of NCDs with the gut microbiome playing a prominent role in between. Routinely used as a preservative in processed meats, inorganic sulfur is metabolized by sulfate-reducing gut bacteria into hydrogen sulfide, previously implicated in the pathogenesis of IBD and CRC.^32,53^ The abundance of sulfate-reducing bacteria in feces was greater in patients with IBD or CRC than in healthy controls, with concurrent increases in fecal levels of hydrogen sulfide.^53^ The excess of colonic hydrogen sulfide has been reported to promote carcinogenesis through DNA damage, reduce the integrity of the mucus layer, and induce epithelial hyperproliferation and immune disorder,^80^ all of which increase the risk for cancer and IBD. Overall, variations in the food ingredients in urbanized dietary habits contribute to the pathogenesis of NCDs, at least partly via their effects on the gut microbiome.

## Urbanized lifestyles

Owing to extended work schedules in most urban societies, the mealtimes of urban residents are shifting to irregularity. The adverse effects of irregular meals on the gut microbiome and host physiology were recently revealed.^81,82^ The gut microbiota can act in response to the feeding rhythm and coordinate the diurnal rhythm in the major histocompatibility complex (MHC)-II-IL-10-epithelial barrier axis in the small intestine by circadian clock.^81^ Disrupted circadian rhythms induced by irregular diet can damage intestinal epithelial barrier integrity, resulting in an extensive antigen influx and exacerbation of Crohn-like enteritis.^81^ A companion study found that regular feeding rhythm induced a rhythmic intestinal epithelial attachment of segmented filamentous bacteria (SFB, a group of gut commensal bacteria).^82^ SFB rhythmically activated group 3 innate lymphoid cells (ILC3) to secret antimicrobial proteins (AMPs) generating day-night variation in resistance to pathogen infection.^82^ These studies suggest an essential role of regular dietary rhythmicity in maintaining intestinal microbiome-epithelial-immune integrity and homeostasis, the disruption of which in urban dietary regimes may predispose individuals to chronic gut dysfunction and disease risks.

Most urban dwellers live a sedentary lifestyle in addition to the aforementioned increased environmental hygiene and cleanliness and the lack of wild/farm exposure. Such a low physical activity lifestyle was found to be associated with increased risks of multiple NCDs and meanwhile alter the composition and metabolism of the gut microbiome.^33,83^ A cross-sectional, observational study (*n* = 82) characterized the gut microbiome of college students with or without moderate-to-vigorous physical activities.^33^ The study discovered that Lachnospiraceae, *Lachnospira*, and Paraprevotellaceae (eminent SCFAs-producers) were more prevalent in college students with greater levels of physical activities, while Enterobacteriaceae and Enterobacteriales (opportunistic pathogens) are more enriched among college students with sedentary behaviors.^33^ Consistently, exercising was also associated with higher fecal concentrations of SCFAs in humans,^83^ which counteract metabolic diseases and autoimmune diseases. The effect of exercise promoting changes in the gut microbiota involves multiple mechanisms of action, including the host release of myokines, the secretion of hormones and neurotransmitters, and the increase of intestinal transit.^84^

Horizontal transmission of gut microbes is limited by smaller family size and fewer livestock/pets feeding in urbanized life. Available evidence suggests that involvement in livestock feeding chores provides rural residents more chances to interact with animal microbiomes through direct contact with animals and their excreta, increasing the gut microbiome diversity of rural residents.^85^ Likewise, a study on familial microbiome similarity found that family members share closely related bacterial genotypes.^86^ This study suggests that large family size and cohabitation in rural societies may increase horizontal transmission of microbes between family members, resulting in a high α diversity and a low β diversity of the gut microbiome, contrasting the signature of urban gut microbiome. Our recent study also showed that infrequent access to shower/bath experienced in rural residents was associated with an enriched gut microbiome and a higher blood high-density lipoprotein (HDL)-cholesterol level,^43^ indicating that rural lifestyle can shape the configuration of the gut microbiome linking to NCDs. Notably, this limitation of gut microbial horizontal transmission may get compounded by preventive or compulsory social isolation due to pandemic. A two-time point analysis of the gut microbiota in healthy humans who underwent quarantine revealed that the intestinal concentration of serotonin (a regulator of several behavioral, mood, and neuroendocrine functions) decreased with the altered gut microbiome in quarantined individuals during 4 ~ 5 months of the quarantine.^87,88^

## Prevalent antibiotic use

With the discovery of antibiotics against infections, the world is experiencing prevalent antibiotic use, especially during pandemics. Both environmental and human-centered studies revealed that urban microbiomes (from environmental and human sampling) have higher abundances of antibiotic resistance genes than in rural microbiomes,^89,90^ implying that antibiotics are commonly used in urban areas and have incurred a genotypic and phenotypic change in both the environmental microbes and the indigenous microbes in humans. Ubiquitous antibiotic use in humans is mostly spurred by fear of pathogens and pandemic legacy. Beyond medical use, low-dose antibiotics, such as feed additives, are commonly used in livestock management to increase weight gain and the mass production of animal-based foods.^91^ The collective use of antibiotics consequently leads to the depletion of naturally occurring microbes in the human gut, posing a gut ecological insult underlying the pathogenesis of NCDs.

In animals, antibiotics contribute to obesity by altering the gut microbiome and the host metabolic activity.^91^ A similar effect was likewise observed in humans.^92^ In a retrospective analysis (*n* = 96), the body mass index (BMI) of patients with infective endocarditis increased significantly after treatment with vancomycin and gentamycin for 6 weeks, where a high concentration of fecal *Lactobacillus* spp. was seen.^92^ A population-based case–control study conducted in Denmark (*n* = 1, 534, 511) indicates that exposure to antibiotics was associated with an increased risk of T2DM.^93^ A single-blinded RCT (*n* = 20) similarly showed that oral administration of vancomycin significantly induced insulin resistance by reducing fecal microbial diversity with a decrease in Firmicutes and a compensatory increase in Proteobacteria.^6^

In addition to increasing the risk of metabolic disorders, antibiotic-induced gut dysbiosis can also contribute to other NCDs. For instance, gut microbiome distortion by antibiotics impaired novel object recognition memory via the gut microbiota-brain axis in mice.^34^ The structural and functional dysbiosis of the gut microbiome induced by antibiotics was found to regulate the expression of cognition-relevant signaling molecules in the brain (up-regulation of neurotrophic factor and down-regulation of N-methyl-d-aspartate receptor subunit 2B, serotonin transporter, and neuropeptide Y system), resulting in cognitive impairment in mice.^34^ Exposure to antibiotics in early life also appears to impact the homeostasis of the gut microbiome and immunity in a long term.^94^ Studies have shown that disruption of immune system development and maturation by antibiotic-induced dysbiosis in early life might result in autoimmune diseases, such as IBD, in later life.^95^ Our earlier study and other studies have consistently shown that antibiotic use could cause opportunistic expansion of certain gut microbes, such as *Candida albicans*, which hinders the assembly and recovery of the gut microbiome.^96^ In mice, administering tylosin (a macrolide antibiotic) and amoxicillin (a beta-lactam antibiotic) after birth for a duration of 5 days exacerbated dextran sodium sulfate-induced colitis in later life.^35^ Such phenotypes could be replicated by transferring antibiotic-perturbed microbiota to GF mice without prior antibiotics exposure, demonstrating that the altered microbiome rather than antibiotics induce exacerbation of colitis later in life.^35^ Overall, antibiotics use has not only changed the genotype and phenotype of individual microorganisms but also changed the composition and functionality of the gut microbiome from a microecology perspective. Alterations that commence in the gut microbiome may ultimately lead to the onset of chronic diseases of systematic nature in conjunction with the direct effects of external factors on the host.

Apart from antibiotics, non-antibiotic drugs deployed in the modern healthcare system are also found to extensively impact the gut microbiome. A high-throughput screen conducted to test 1,197 US Food and Drug Administration (FDA)-approved non-antibiotic drugs against 40 gut bacterial species found that 24% of these compounds inhibited the growth of at least one bacterial strain.^97^ This data suggests that with the advancement in the development and deployment of medications, the effects of drugs on the gut microbiome should be cautioned.

## Early life exposure in an urbanized environment

Exposure to the gut microbiome in early life is conducive to the development and education of the host immune system.^98^ Defects in lymphoid tissue development observed in GF animals suggest that early-life microbial colonization in the gut is important in the development of mucosal and systemic immunity.^99^ The transcriptional profile in the jejunum and colon of GF mice conventionalized during adult life is different from specific pathogen-free (SPF) mice, suggesting that there is a narrow “critical window”, to educate the host immune system via the gut microbiome.^100^ A multitude of studies have shown that dysbiosis of the gut microbiome in early life is associated with later-life diseases, such as allergy diseases, metabolic diseases, autoimmune diseases, and neurological disorders.^95,101–104^

The delivery mode can strongly affect the first major microbial exposure and colonization in neonates.^38,105^ The gut microbial diversity of vaginally born neonates was higher than those born through cesarean section, and 72% of microbial species from vaginally born neonates matched the species found in the feces of their mothers, suggesting vertical mother-to-neonate transfer.^38^ In contrast, the first colonizers of cesarean-delivered infants come from the surrounding environment during delivery.^38^ Lower overall microbial diversity, lower abundances of probiotics (*Bifidobacterium*, *Bacteroides*, and *Lactobacillus*), but higher abundances of *Enterobacter*, *Haemophilus*, *Staphylococcus*, and *Streptococcus*, were found in cesarean delivered infants compared to vaginally delivered infants.^105^ Cesarean delivery rates are generally higher in urban areas than in rural areas.^106,107,108^ The overuse of cesarean delivery in urban areas, especially in Brazil, China, and the US, was associated with dysbiosis of the gut microbiome in neonates and an enhanced incidence of NCDs, particularly asthma.^109,110^ A prospective study conducted in south-eastern Michigan in the US (*n* = 298) found an association between a specific gut microbiome feature of neonates and a high risk of asthma:^37^ both cesarean-delivered infants and asthma patients have a low relative abundance of *Bifidobacterium* in their gut microbiomes.^105^ This association can be explained by the fact that gut microbiome dysbiosis in neonates could promote CD4^+^ T cell dysfunction underpinning the pathogenesis of asthma.^37^ The *ex vivo* culture of human adult peripheral T cells with sterile fecal water from neonates with the highest risk of asthma increased the compartment of CD4^+^ IL-4^+^ cells (pro-inflammatory) yet reduced the proportion of regulatory T cells (Treg cells, anti-inflammatory).^37^ In addition, a 20% increase in the risk of childhood onset of T1DM was reported in a meta-analysis of cesarean-delivered children,^111^ also associated with the reduced abundance of *Bifidobacterium* in cesarean-delivered children.^112^

Breastfeeding is another major factor that shapes the composition of the newborns’ gut microbiome in the early-life window. Diverse probiotic microbial strains from the genera *Bifidobacterium*, *Lactobacillus*, and *Streptococcus* can transfer from the mother’s gut to breast milk via the gut-mammary axis.^113^ Together with the milk oligosaccharides (a source of MACs), microbes in breast milk are ingested and colonized in the infants’ gut.^114^ In addition, IgA in breast milk can govern the development of retinoid-related orphan receptor-γ (RORγ^+^) Treg cells and microbiomes in the gut of infants, tuning the immunologic and microbial profiles in infants. Given the importance of breastfeeding, the extensive use of infant milk formula in urban areas compared to rural areas^115^ decreases the vertical transmission of the microbiome from mother to baby, especially beneficial microbes, such as *Lactobacillus* spp. and *Bifidobacterium* spp., as well as the transmission of immunoglobulins which plays an instrumental role in shaping the gut microbiome and immunity of infants.^38^ On the other hand, one caveat is that vertical microbial transmission from urban-dwelled mothers to infants might not be as beneficial, considering that urban dwellers may already harbor a (pre-)dysbiotic gut microbiome which upon transmission may increase the risk for NCDs in the next generation.^116–118^ To illustrate, maternal pre-pregnancy overweight has been shown to increase the risk of childhood overweight, mediated by the vertical transmission of Firmicutes.^116^ An overweight mother can also impact the gut microbiome and health outcomes of her children via the composition variation of breast milk. Breast milk of overweight mothers contains higher levels of *Staphylococcus* and lower levels of *Bifidobacterium*, both of which have been associated with allergies, inflammatory diseases, and metabolic diseases.^117,118^

## Harnessing the gut microbiome to counteract NCDs in urbanized societies

**Figure 4.**
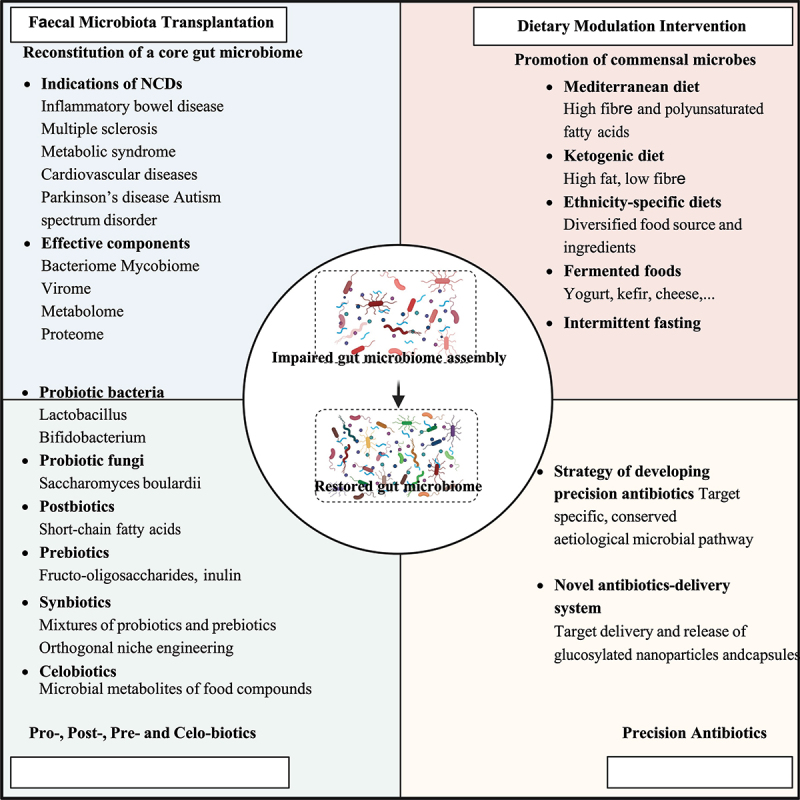
Harnessing the gut microbiome to counteract NCDs.

In recognition of the prominent roles of the gut microbiome in modulating human health and disease risks, a myriad of therapeutics inspired by or targeting the gut microbiome is under development (Figure 4). These measures may reduce NCDs by eradicating the etiological gut microbial species to NCDs and/or introducing beneficial keystone species, and represent avenues applicable and accessible to global populations.

## Faecal microbiota transplantation

Fecal microbiota transplantation (FMT) is an innovative treatment for diseases that are associated with gut dysbiosis, by transferring the fecal suspension containing the microbiome from a healthy donor into the GI tract of a recipient with disease.^119,120^ FMT has been used to treat *Clostridioides difficile* infection, with approximately an 85–90% response rate and a successful restoration of the gut microbiome in patients^121^. In consideration of the intimate connection between IBD and gut dysbiosis, FMT has been regarded as a potential therapeutic for IBD.^120,122,123^ A meta-analysis evaluating 6 RCTs found that FMT resulted in significant clinical and endoscopic remission versus placebo (odds ratio, 4.11; 95%CI, 2.19–7.72).^122^ Compared to glucocorticoid (a commonly used IBD medication), FMT showed a comparative efficacy to induce remission in active mild-to-moderate UC, but with fewer adverse events.^123^ Although current studies on FMT in CD enrolled a limited number of participants, the data are encouraging implying a therapeutic potential of FMT.^120^ According to a meta-analysis of 12 studies, FMT resulted in clinical remission and clinical response in 62% and 79% of patients with CD, respectively.^124^ In addition, FMT was reported to significantly decrease the rates of clinical relapse of CD patients with anti-tumor necrosis factor (anti-TNF) failure.^125^ Several RCTs also showed that FMT was effective in treating metabolic diseases, increased insulin sensitivity in patients with metabolic syndrome,^126^ and decreased the plasma levels of TMAO in patients with CVD.^127^ Furthermore, the benefits of FMT in Parkinson’s disease and CRC were also explored in mice.^128,129^ These studies suggest that FMT holds a promising prospect in reducing NCDs by resetting the gut microbiome underlying a disease.

One caveat of the application of FMT in clinical practice is that the efficacy is highly heterogeneous across different individuals.^126,127,130^ One explanation is that the efficacy of FMT relies, at least partly, on the composition of the fecal microbiome from the donor or that of the recipient, as demonstrated in our earlier studies.^131,132^ We found that a high abundance of fecal *C. albicans*, either in the donor or in the recipient before treatment, reduced the efficacy of FMT.^132^ In contrast, a higher fecal viral microbiome richness of *Caudovirales* bacteriophages from donors was associated with a favorable clinical outcome.^131^ These data suggest that screening and selection of appropriate donors and proper donor-recipient pairing might enhance FMT efficacy. The overall lesson on FMT is that there is no one-size-fits-all donor, especially if the donors are urban individuals. Future precision FMT may involve rational selection and design of the donor microbiome according to the recipient microbiome. On the other hand, FMT carries a risk for transmission of under-detected or emerging pathogens; therefore, developing a components-definite, purified, and pathogens-free microbial cocktail may serve as an alternative and refined microbiome therapeutic approach. SER-109, an investigational oral microbiome therapeutic composed of live purified Firmicutes bacterial spores, has been approved by the FDA and showed a safety and efficacy profile in CDI.^133^ Meanwhile, SER-287 (Firmicutes spores) was found to induce clinical remission in patients with UC in the phase 1b trial.^134^ Overall, both FMT and refined microbial-consortia represent promising microbiome-based treatment modalities for combating NCDs.

## Dietary modulation and intervention

Diet is considered a mild but highly effective modulator of the gut microbiome composition and host health. A variety of characteristic diets, regional or ethnic, are demonstrated to be associated with lower risks of NCDs.^135–137^ Of them, the Mediterranean diet is recognized as an exemplary mode of healthy eating. This dietary pattern is distinct from the typical western diet, emphasizing the intake of highly complex carbohydrates, polyunsaturated fatty acids, and bioactive compounds with antioxidative properties.^136^ A study of 27 healthy individuals revealed that the Mediterranean diet can attenuate the increase in Firmicutes:Bacteroidetes ratios induced by western diet.^138^ A high abundance of probiotic bacteria, *Prevotella* spp., *Clostridium* cluster XIVa, *Faecalibacterium prausnitzii*, and *Bifidobacterium* spp. was found in individuals consuming the Mediterranean diet.^139^ Moreover, metabolomic profiling of Mediterranean diet-consuming individuals revealed a high concentration of SCFAs in feces, which have favorable effects on intestinal barrier integrity, implying the potential to counteract numerous chronic diseases.^140^

Ketogenic diet containing high fat, moderate protein, and very low carbohydrate is another characteristic diet. It has been used to treat refractory epilepsy in children for decades,^141^ and was, in the last decade, shown to have immense beneficial effects on many chronic diseases, including metabolic diseases, autoimmune diseases, and cancers.^142,143^ These findings renewed interests in the ketogenic diet, making it a popular diet mode in urban societies. Ketogenic diet also exhibits therapeutic potential against neurologic disorders via the microbiota-gut-brain axis, including Parkinson’s disease, Alzheimer’s disease, autism spectrum disorders, and multiple sclerosis.^144,145^ Moreover, ketogenic diet is recognized as a fad diet for weight loss and alleviating IBD.^135^ A recent observational study in obese subjects (*n* = 15) reported a sharp decrease in the fecal abundance of obesity-associated microbes, Lachnospiraceae, *Eubacterium hallii*, *Pseudomonas*, and *Blautia*, concurring with a significant reduction in BMI in obese patients after a 12-week ketogenic diet intervention.^135^ A recent mechanistic study reported that the ketogenic diet alleviated colitis by reducing the production of RORγt^+^CD3^−^ ILC3 and related inflammatory cytokines (IL-17α, IL-18, IL-22, and CCL4) in mice, which was attributed to the modification of gut microbiota (increase in *Akkermansia* and decrease in *Escherichia*/*Shigella*).^142^

Beyond the Mediterranean diet and ketogenic diet, our recent population-wide study conducted on healthy Chinese (*n* = 930) found that ethnicity-specific diets were associated with the presence of certain gut species as well as a low risk of some NCDs, such as IBD.^43,146^ For instance, the ethnic Chinese Zang-specific diet with butter milk tea consumption was associated with the gut presence of microbial lineages from the *Penicillium* and *Naumoyozyma* genera, which mirrors a substantially low disease prevalence of IBD in ethnic Zang Chinese. In addition, fermented foods, such as yoghurt, kefir, cheese, fermented vegetables, nattō, bread, wine, and beer, are a type of food containing a high number of probiotic species and their metabolites.^147^ A recent 17-week randomized, prospective study (*n* = 39) found an increased gut microbiota diversity and decreased markers of host inflammation in healthy adults consuming a large number of fermented foods in their diet.^137^ However, the effects and applications of these characteristic diets as well as the functions of the under-studied gut microbes induced by these diets await elucidation.

Apart from “what to eat”, “how to eat” is also important to shape the gut microbiome and counteract NCDs. Intermittent fasting and time-restricted eating are dietary programs that manipulate the timing of eating occasions by utilizing short-term (longer than overnight) fasting. This dietary program has been shown to induce the browning of white adipose tissue by reshaping the gut microbiota and is shown to be effective in weight control.^148^ Moreover, intermittent fasting can also ameliorate autoimmune diseases. A murine study found remission of IBD and intestinal barrier regeneration after intermittent fasting.^149^ Fecal transplants from intermittent-fasting-treated donor mice, characterized by an expansion of the probiotics Lactobacillaceae and Bifidobacteriaceae, attenuated disease phenotype in recipient mice with colitis, indicating that the gut microbiome mediates the benefits of intermittent fasting.^149^ A similar outcome was reported by a study in mice with multiple sclerosis, where the gut microbiome was reshaped by intermittent fasting following which the clinical course and pathology of multiple sclerosis were ameliorated.^150^ In general, modulating the profile of dietary components and the timing of eating represent two scalable and amenable strategies to treat NCDs and exploit the gut microbiome for health.

## Probiotics, postbiotics, celobiotics, and synbiotics

Probiotics are live microorganisms providing health benefits and counteracting NCDs through various mechanisms, such as regulating intestinal microbiota,^151^ inhibiting pathogens,^152^ maintaining the epithelial barrier,^153^ activating host immune,^154^ and modulating host metabolism.^155^ Gut microbial species that are decreased in urban residents meanwhile that can counteract NCD pathogenesis should be developed into probiotics for treating NCDs. For example, VSL#3, a high-concentration probiotic bacteria mixture, has been proven to be effective in UC by several RCTs.^156,157^ Beyond bacteria, fungi can also serve as probiotics conferring a health benefit on the host. One month of administration of the probiotic yeast *Saccharomyces boulardii* in mice with obesity and T2DM ameliorated the gut microbiome, resulting in reduced obesity, hepatic steatosis, fat mass, and inflammation.^158^

The effect of probiotics can be partially mediated by their metabolites, which are now used as postbiotics to improve human health and treat NCDs. SCFAs, such as acetate, propionate, and butyrate, produced by *F. prausnitzii*, *A. muciniphila*, and several anaerobic commensal bacteria, are a class of extensively investigated postbiotics. Butyrate is a preferred energy source for colonic epithelial cells that are engaged in maintaining gut barriers and regulating immune and metabolic homeostasis.^59^ Enemas of butyrate resulted in clinical and histological improvement in active UC patients, by inhibiting histone deacetylase (HDAC) and the translocation of NF-κB in lamina propria macrophages in tissue sections.^159^ Likewise, in DSS-treated mice, butyrate enemas reduced colonic mucosal damage and inflammatory cytokines (IL-6, TNF-α, and IL-1β).^160^ Not only can propionate stimulate colonic goblet cells to secrete glucagon-like peptides (GLP) but it can also inhibit the synthesis of cholesterol and the production of new fat in the liver in rats.^161^ Another type of microbial metabolites that can be used as postbiotics are secondary bile acids, which directly regulate certain intestinal immune cell populations.^162^ Of them, 3-oxolithocholic acid can inhibit the differentiation of Th17 cells by binding to the key transcription factor RORγt, while isolithocholic acid increased the differentiation of Treg cells through the production of mitochondrial reactive oxygen species (mitoROS) and up-regulated expression of FOXP3,^163^ implying the potential of restoring the gut immune-homeostasis and treating IBD.^164^ Apart from producing beneficial metabolites, probiotics can also provide therapeutic potential by liberating celobiotics. Celobiotics are bioactive compounds that are liberated from indigestible food components through the actions of one or more microbial enzymes (rather than being synthesized).^60^ A recent study found that gut microbes, such as *Bacteroides ovatus* and *Parabacteroides distasonis*, could liberate a celobiotic *N*-methylserotonin from orange fiber, a generally discarded by-product of orange juice manufacturing.^60^ The liberated *N*-methylserotonin reduced adiposity and improved host metabolism in mice.^60^ This study presents a primer to utilize celobiotics or paired combinations of bioactive components and their “microbial miner”, to subvert the unfavorable effect of food over-processing and manufacturing on host health during urbanization and industrialization.

As it is often difficult to achieve durable engraftment of probiotics without the use of antibiotics,^165^ probiotics are usually proposed to be supplied with prebiotics (substrates selectively utilized by gut microorganisms, such as MACs), together known as synbiotics.^166^ Prebiotics are employed to enhance the survival and colonization of probiotic microbes by providing a nutrient niche. On the other hand, probiotics ensure that the gut microbiota can metabolize prebiotics to generate beneficial compounds for the host or the co-occurring microorganisms in the gut.^166^ Multiple RCTs have shown that synbiotics combinations were effective in treating certain NCDs. A mixture of the probiotics *Lactobacillus* and *Bifidobacterium* supplemented with the prebiotics fructo-oligosaccharides or inulin was found to ameliorate IBD^167^ and obesity.^168^ Despite the beneficial effects of these prebiotics, the arsenal of prebiotics remains to be substantially expanded as the nutrient and metabolic preferences of the large diversity of gut microbes are largely unknown to date. Orthogonal niche engineering is a recently developed approach to test for the proper substrates for the targeted probiotic microorganisms and to maintain a controllable, long-term colonization of the microorganisms of interest. For instance, Shepherd et al. transferred the porphyran utilization locus into a gut commensal strain *B. ovatus* NB001 to enable it to utilize porphyran, a component of seaweed.^169^ This approach allowed for fine control of colonization and abundance of this recombinant *Bacteroides* strain in the murine gut by varying the dosage of the marine polysaccharide porphyran.^169^ Through this strategy, orthogonal niche engineering in conjunction with probiotics biosynthesis offers a novel combinatorial approach to reintroduce and maintain the beneficial microbes that are depleted or functionally compromised during urbanization.

## Precision antibiotics

During clinical application, low-dose antibiotics may fail to clear disease-causing microorganisms and evoke antimicrobial resistance, while overdosing on antibiotics may lead to dysbiosis of a broad spectrum of gut microorganisms. Therefore, developing precision antibiotics that only target the microbial species that cause NCDs while leaving other microbial members in the gut largely spared becomes an urgent unmet need. Metagenomic analysis of the gut microbiome of patients with IBD identified the molybdenum-cofactor-dependent metabolic pathways of the gut microbes as a signature of inflammation-associated dysbiosis in IBD.^170^ Guided by the target microbial pathway, the authors next devised a tungstate-treatment strategy to specifically inhibit the microbial molybdenum-cofactor-dependent respiratory pathways.^171^ Following administration, the compound tungstate attenuated colitis and selectively suppressed the dysbiotic expansion of Enterobacteriaceae while exhibiting a minimal effect on the overall gut microbiota.^171^ This example represents a rationally designed case of precision antibiotics to treat IBD. Future efforts to counter NCDs-related gut dysbiosis may refer to this strategy by selectively targeting etiological microbial pathways to the pathogenesis of NCDs.

The antimicrobial effect of antibiotics depends on the drug concentration at the target site, hence optimizing the dosing and release site of antibiotics within the host is critical for combating NCDs. To this end, novel antibiotics-delivery technology and system are in development to minimize the collateral destruction to the holistic intestinal microecology. Zhang et al. recently developed an encapsulation system that encased antibiotics into glycosylated nanoparticles with a positive charge, which can be readily absorbed in the proximal small intestine through the intended interaction between glucose in the nanoparticles and the abundantly expressed sodium-dependent glucose transporter 1 in the intestinal epithelium.^172^ This glucose-decorated encapsulation can significantly reduce the accumulation of antibiotics in cecal and fecal contents, therefore reducing perturbations to the colonic microbiota.^172^ Although it is a daunting challenge, such strategies can be adopted and modified to target tumor sites in GI cancers, to aim for controlled release at the inflamed sites for treating GI inflammations or aim for controlled release upon sensing the disease-causing microorganisms. Overall, target-orientated selection of antibiotics along with the rational design of antibiotics-delivery techniques would substantially enable precision-killings of NCD-causing gut microbes.

## Conclusions and future directions

With advancements that unveil the intricate relationship between the gut microbiome and NCDs in the age of urbanization, identifying the keystone species that are virtually etiological or therapeutic to an interrogated NCD is critical. However, there remain a series of challenges and pitfalls in the field: 1) while a lot of studies showed associations between alterations in the gut microbiome and particular NCDs in humans, only a minority of studies could establish causality between a gut microorganism of concern and an NCD; 2) on the other hand, many microbial species identified to cause an NCD in pre-clinical models (such as mice and rats) cannot be replicated in humans, which impairs translating the findings from animals to humans; 3) on a similar note, clinical trials with the same intervention may result in equivocal outcomes due to individualized responses of the various gut microbiomes in humans, limiting the translation from bench to bedside; 4) it is an expensive challenge to identify environmental and dietary risk factors in urban societies that are actually genuine contributor to NCDs and gut microbiome dysbiosis, due to the substantial heterogeneity of population characteristics and confounding factors; 5) among the large consortium of gut microbes, ranging from bacteria, fungi, archaea to viruses/phages, it is daunting to discern functional and actionable microbes to treat NCDs, so is to expand the toolkit to modify them.

With these knowledge gaps and challenges being addressed, we will witness a bright future harnessing the gut microbiome to counteract NCDs. Meanwhile, the enhanced sanitation and social isolation measures due to unforeseen outbreaks of infectious diseases, such as the raging COVID-19, Monkeypox, and Polio, can further amplify “the disappearing gut microbiome during urbanization” in our era. This presents as a legacy of the decrease in the diversity of environmental microbiomes and human microbial exposures. Therefore, future city planning should take into consideration strategic city partitioning and diversification of the environmental microbiomes from the ecology perspective, to facilitate salutary microbiome exchanges between the environment and humans, and between humans.
